# Rapid response in health technology assessment: a Delphi study for a Brazilian guideline

**DOI:** 10.1186/s12874-018-0512-z

**Published:** 2018-06-08

**Authors:** Marcus Tolentino Silva, Everton Nunes da Silva, Jorge Otávio Maia Barreto

**Affiliations:** 1 Faculty of Medicine, Federal University of Amazonas, Manaus, Brazil; 2 Faculty of Ceilândia, University of Brasilia, Brasília, Brazil; 3 Oswaldo Cruz Foundation, Brasília, Brazil

**Keywords:** Health technology assessment, Rapid response, Rapid reviews, Consensus, Delphi

## Abstract

**Background:**

Rapid response in health technology assessment is a synthesis of the best available evidence prepared in a timely manner to meet specific demands. We build a consensus among Brazilian specialists in health technology assessment to propose guidelines for the development of rapid response.

**Methods:**

Based on a systematic review that proposed eight methodological steps to conduct rapid response, we applied a modified Delphi technique (without open questions in the first round) to reach consensus among Brazilian experts in health technology assessment. Twenty participants were invited to judge the feasibility of each methodological step in a five-point Likert scale. Consensus was reached if the step had 70% positive approval or interquartile range ≤ 1.

**Results:**

The achievement of consensus was reached in the second round. Between the first and the second round, we scrutinized all points reported by the experts. The Delphi panel reached consensus of eight steps: definition of the structured question of rapid response (with a restricted scope); definition of the eligibility criteria for study types (preferably systematic reviews); search strategy (language and data limits) and sources of information (minimum two); selection of studies (independently by two responders); critical appraisal of the included studies and the risk of bias for the outcomes of interest; data extraction from the included articles; summary of evidence; and preparation of the report.

**Conclusions:**

The guidelines for rapid response in health technology assessment may help governments to make better decisions in a short period of time (35 days). The adoption of methodological processes should improve both the quality and consistency of health technology assessments of rapid decisions in the Brazilian setting.

## Background

Health managers are increasingly pressured to make quick decisions on the use of health technologies [1]. The information retrieval methods available in the health manager’s perspective have been the subject of discussion [2]. Regardless of the approach adopted, a hierarchy of evidence directs the process to the most reliable publications [3].

In this scenario, systematic reviews are prioritized by their ability to disseminate the information on a well-structured question [4]. However, the time and structure necessary for the creation of such systematic reviews could be impractical in health management [5]. Usually, a systematic review requires between 6 months and 2 years to be finished. On the other hand, a large body of systematic reviews—larger than that of clinical trials—has been produced [6]. Thus, systematic reviews may be used as first sources of information for answers about the use of health technologies.

Health technology assessment agencies are adopting rapid reviews to support decision in a timely manner [7]. Experiences from Canada [8] and Scotland [9] reveal that transparency and intensive user feedback increase the responsiveness. They also report a broad range of topics: since health service utilization to drugs prescription. Normally, the shortcuts adopted show similar results with systematic reviews. These shortcuts include limited sources of information and critical appraisal tools for systematic reviews [10–12] and primary studies [13–16].

Such elements reinforce the need for a standardization of the steps for rapid reviews within the framework of health management [2]. Some institutions have already defined their strategy [17], like Brazil in the development of the Methodological Guideline ‘elaborating mini health technology assessment’ [18] and the World Health Organization [19]. However, information sources are sometimes inaccessible, or strategies can be time consuming (the Brazilian guideline suggests three months of preparation).

In this scenario, we build a consensus among Brazilian specialists in health technology assessment to propose guidelines for the development of a rapid response to be carried out in 35 days. This time frame was defined by the Ministry of Health, because their process of decision making about introduction of a health technology should be completed by end of 180 days.

## Methods

### Design and settings

This is a process of consensus performed by a modified Delphi technique sent by email [20]. In the first semester of 2017, researchers in health technology assessment (HTA) from Brazilian universities or research institutions were contacted.

The Delphi method consists of steps of semi-structured questionnaires applied remotely [21]. The items of the questionnaires are composed by consensus statements in the first round. On this basis, every item receives a numbered judgment scale and a comment space. Depending on the score obtained among several specialists, the affirmative is defined as consensus or is revised/corrected for a new round of questionnaire application. The steps are repeated until consensus is achieved in all statements.

In the present study, the Delphi technique was modified: the affirmatives in the first round are based on the literature review [2] rather than open questions to the participants.

### Participants

The Delphi technique does not require the inclusion of a random sample of specialists to ensure representativeness [20]. It is recommended that participants be homogeneous in their characteristics and that a sample size between 10 to 15 participants is enough to generalize the consensus [20]. So, at first—and considering possible losses—20 specialists in HTA were invited to participate in the consensus.

We identified 25 Brazilian researchers across the country who met our inclusion criteria (five refused to participate): (i) knowledge and practical experience in HTA in the last three years; (ii) ability and willingness to contribute; (iii) free time to dedicate to completing the questionnaires; (iv) good written communication skills; and (v) a PhD academic degree. All participants were invited by e-mail or by telephone. We balanced the representativeness of regions over the country. These criteria for selecting experts ensured that all participants had prior training and experience in review synthesis and were considered suitable for making accurate judgements. The experts worked anonymously and independently of each other.

### Processes

A semi-structured questionnaire composed of eight items was used based on a previous study [2, 22]. Each item makes up the content’s recommendation of the future guidelines for the development of rapid response in HTA. For the judgment, a Likert scale was chosen, assigning one point to total disagreement on the item and five points to full agreement. We have also provided space for comments for every item. A letter with instructions was sent with a fictional example that included a Likert scale and a comment box filled. Comments were open, independently of agreement or disagreement.

The questionnaire has also covered some attributes from the panel experts, such as gender, age, education, function, main activity at work, experience in HTA and the place of residence. The questionnaires were sent by email to participants, who had 10 days to return the information in each round.

### Statistical analysis

Questionnaire items were analyzed individually. In each round, consensus was defined if the item has [20]: (i) 70% positive approval or (ii) interquartile range ≤ 1. Positive approval is calculated by the following formula based on the individual item Likert scores across respondents:

$$ Approval=\frac{\left(\sum obtained\ score\right)-\left(n\times minimum\ possible\ score\right)}{\left(n\times maximum\ possible\ score\right)-\left(n\times minimum\ possible\ score\right)}x100 $$

n: number of participants

The interquartile range was obtained by the difference between the third quartile and the first quartile. All items that presented an approval rate lower than 70% were modified according to the comments received by the experts for a new Delphi round. When an item reached consensus on its inadequacy (below 70% and interquartile range ≤ 1), the item was completely reformulated. We estimated three Delphi rounds to obtain consensus.

## Results

Twenty experts completed the first round. Most of them were physicians (60%) or pharmacists (15%). Females represented 70% of the expert panel. The mean age was 50.1 years old (standard deviation 10.0), and the mean experience in HTA was 12.1 years (standard deviation: 7.1). Most of the experts work in universities (50%), research institutes (25%) or hospitals (20%), and their main activities are teaching (50%) or research (40%).

Table 1 presents the consensus results for each Delphi round. Between the first and the second round, we wrote an introduction about the rapid response, which achieved 75% of consensus. Below we described each item of the rapid response, including the number of professionals involved and length of days for each step.

**Table 1:** Consensus results in the first and second round

Steps	Description	First round (*n* = 20)		Second round (*n* = 16)	
		Consensus (%)	IQR	Consensus (%)	IQR
1	Definition of the structured question	84.2	1.0	84.4	1.0
2	Definition of the eligibility criteria for study types	75.0	2.0	73.4	1.0
3	Search strategy and sources of information	56.6	2.0	76.6	1.0
4	Selection of studies	59.2	2.0	85.9	1.0
5	Quality assessment of the included studies and the risk of bias for the outcomes of interest	68.4	1.0	76.6	1.0
6	Extraction of data from the included articles	85.5	1.0	90.6	1.0
7	Summary of evidence	81.6	1.0	84.4	1.0
8	Preparation of the report	71.1	1.0	81.3	1.0

*IQR* interquartile range

### Step 0. Why rapid response in health technology assessment?

Rapid response in the health technology assessment is a synthesis of the best available evidence prepared in a timely manner to meet specific demands. These documents weigh the methodological quality of scientific studies. Thus, professionals with experience in systematic reviews are qualified for this activity. The main aim of rapid response is to indicate the best scientific evidence available on the consequences of a health technology. It is outside the scope of the rapid review to provide recommendations, since additional elements involved in decision-making are difficult to consider in a short period of time. To increase the reproducibility and transparency and to minimize potential biases, it is suggested that the rapid response protocols (steps 1 to 3) be reviewed by an editor or supervisor. Such protocols should be registered in a separate repository.

To meet the request of the user, the product is expected to be elaborated within 35 days. On the other hand, according to the soliciting party’s interest and complexity of the issue, this time limit may be flexible. It should be noted that consulting important documents, such as clinical guidelines and evidence-based synopses, will facilitate the process. Finally, the scope of the rapid response should be agreed on by the soliciting party for greater adherence to the product.

### Step 1. Definition of the structured question (1 business day; 2 professionals)

Once the scope is defined by the soliciting party, the initial stage of rapid response is the definition of the structured question. Rapid response should have limited scope and address a single, specific issue. The question should be clarified and preferably adopt the PICO (Population, Intervention, Comparison, Outcomes) structure. When applicable, the population of interest will consider potential subgroups. Some information about the intervention or the technology evaluated improves its specification, such as: (i) registration of technology in the Brazilian National Health Surveillance Agency (ANVISA), if applicable; (ii) stage of incorporation into the Unified Health System (SUS), which is the public health system in Brazil; and (iii) presence in national clinical protocols. The comparator, when available, will reflect the technology already available within the analyzed context. Clinically relevant outcomes for the target population will be prioritized, which may be reviewed by the editor or supervisor.

### Step 2. Definition of the eligibility criteria for study types (1 business day; 1 professional)

At this stage, the reviewer will specify which studies to consider in preparing the rapid response. Such criteria must be in accordance with the type of question (treatment, diagnosis, prognosis, etc.). In most cases, secondary studies will be prioritized, specifically the ones that evaluated the efficacy and safety of the target technology analyzed, with emphasis on the reports of health technology assessment and systematic reviews/meta-analysis. In some scenarios, there will be a need to retrieve primary studies (randomizaed clinical trials, cohorts, case-control studies) due to the absence or need for an update in secondary studies (health technology assessment reports, systematic reviews, guidelines). When considering primary studies, a hierarchy of evidence will be considered according to the type of rapid response structured question. Date and language limits may also be specified, depending on the amount of studies and the ability of the team.

### Step 3. Search strategy and sources of information (2 business days; 1 professional)

This step consists in defining two parameters that allow the reproducibility of the rapid response: (i) terms to be used in databases according to the question in step 1; and (ii) search locations of the studies prioritized in step 2. Preferred terms are those used in the cataloging of biomedical studies (available in DeCS and MeSH). In the absence of this record, it is suggested to use the most commonly used synonymy or common denomination. Depending on the structure available, an expert will evaluate the strategy adopted. At least two sources of information must be consulted. Meta-search engines, such as NHS Evidence Search, tend to minimize the search time since there are filters for secondary and primary studies. It is suggested that Medline (via PubMed, Clinical Queries) is consulted to search for systematic reviews and evidence of potential primary studies. In some scenarios, it will be necessary to seek unusual sources (depending on available resources) or consult experts, who must be specified and justified.

### Step 4. Selection of studies (5 business days; 2 professionals)

In the best-case scenario, it is suggested that the search results are evaluated by two reviewers. Normally, the selection starts with titles and abstracts and moves forward to the reading of the full texts. Disagreements are resolved by discussion. The process tends to be faster through the use of specific software, such as reference management or electronic spreadsheets. These tools require importation of file results (in RIS, CSV, XML formats, etc.) and will reduce duplicate records.

### Step 5. Critical appraisal of the included studies and the risk of bias for the outcomes of interest (5 business days; 2 professionals)

This step consists of two phases: (i) assessment of methodological quality of the included studies; and (ii) analysis of the risk of bias on the effect of intervention in the analysis for the outcomes of interest. It is suggested that this step is performed by a professional and that it is reviewed by another. The data must be arranged in tables. At first, it is possible that secondary or primary studies are included. In case the secondary study is included, using the tools AMSTAR [11], ROBIS [12] or similar is recommended for its appreciation. In the circumstance of including primary studies, its appreciation is suggested through the Cochrane Collaboration [13] tool for randomized controlled trials or New-Castle-Ottawa for observational studies [14]. Other critical evaluation tools are suitable [15], such as the diagnostic run through QUADAS [16], provided that they are justified.

The second stage is the assessment of potential risk of bias in the measurement of intervention performance for the outcomes of interest. The GRADE [23] approach is suggested for this purpose. Initially, the study delineation is identified in the research subjects (randomized controlled trial or observational study). Then, the potential problems of the studies identified are listed (according to critical evaluation of a systematic review of good quality or according to the judgement of the reviewer). Subsequently, inconsistency (heterogeneity), indirect evidence (population, intervention, comparator or outcome) and uncertainty (confidence intervals) are weighed. Thus, quality of evidence will be set as high (new studies are unlikely to change the estimates found), moderate (new studies may modify the estimates), low (new studies will modify the estimates) or very low (uncertainty as to the effect of the intervention on the outcome).

### Step 6. Extraction of data from the included articles (10 business days; 2 professionals)

This step involves the extraction of information from the studies through the development of tables. Thus, tabulation of the information regarding author, year, context (location and dates), delineation, research subjects (inclusion and exclusion criteria), sample size, evaluated interventions and outcomes is suggested. Such activities will be carried out by a professional and will be reviewed by a second one.

### Step 7. Summary of evidence (5 business days; 1 professional)

The rapid response results will be presented in tables containing the results by outcome. The following data will be tabulated: (i) the outcome; (ii) measurement of effect in relation to the comparator (relative risk, odds ratio, difference of averages, etc.) with a 95% confidence interval; (iii) number of participants and studies; and (iv) quality of evidence (reliability, according to step 5). If necessary, additional comments may be registered on the table or in the form of a note.

### Step 8. Preparation of report (5 days; 1 professional)

The rapid response report will include the following: Title (technology X for indication Y); Executive Summary: contains information about: technology, indication, question, evidence recovered, main findings and conclusions (up to 400 words); Context, including the following key aspects: (a) record of technology in regulatory body, (b) stage of incorporation to the health system, and (c) technology insertion into national clinical protocols (up to 400 words); Question: population intervention, comparator, outcome; Methods: description of the procedures performed, with emphasis on (i) sources of information, search strategy, results found, and date of completion; (ii) the selection process; and (iii) critical evaluation (up to 400 words); Evidence: description of retrieved studies (objective, methods, preliminary and/or main findings, limitations) and respective assessment of methodological quality (summary table); Summary of results: description of results of interest to the Rapid Response structured question (up to 600 words); Conclusion: global synthesis of results on the issue (up to 200 words); References (with links to access, where available): Vancouver standard; Identification of those responsible for the preparation: name, title, affiliation and contact; Declaration of potential conflicts of interest of those responsible for the preparation; Link to access the rapid response protocol used.

## Discussion

### Main findings

We have developed a rapid response standard to be carried out in 35 days with eight steps, based on two rounds of a modified Delphi approach. All items reached consensus between 73 and 91%. Important insights from experts were included, like two reviewers in the selection of studies. This is the first consensus to our knowledge on methodological requirements on rapid response for HTA to endorse a decision-making process.

### Strengths and limitations

Our study supports a previous synthesis of shortcuts for rapid response [2, 22] with a recognized method of consensus [20] to generate potential standards for governments. A key strength was the wide range of expertise (professors, researchers and health managers, from public and private sectors) within our panel, though we acknowledge that we may not have encompassed all possible perspectives. On the other hand, the fact that all steps were confirmed in the first round indicates their expertise in the field. Between the first and the second round, we considered the comments and revised the rationale for each step. Despite our emphasis during recruitment and rounds, four participants dropped-out between the rounds.

Previous studies identified numerous definitions and process for rapid reviews in HTA agencies [7, 19]. We opted by ‘rapid response’ because the Brazilian Ministry of Health has a guideline for rapid HTAs [18] and a demand for a more express return. These nomenclatures make it difficult to compare methods and results of different HTA processes [24]. Our emphasis is that each rapid response will increase the transparency of their methods [19, 25].

### Interpretation

Three steps were modified between the first and second round: (i) search strategy and sources of information; (ii) selection of studies; and (iii) quality assessment of the included studies and the risk of bias for the outcomes of interest. In the first step, the modifications reflect that important studies can be found within a narrow sum of databases [26] and the poor impact provided by gray literature [27]. In the second step, we made corrections about ascertainment of screening records that should be made by two professionals, whenever possible [28]. In the third step, we expanded the possibilities of critical appraisal tools [11–16] and the use of the GRADE approach [29] to provide transparency. All modifications were made based on the panel commentaries and the literature review.

An international survey indicates that decision makers can accept some concessions in validity in exchange for an accelerate synthesis of the evidence [30]. However, they also expect the results to be reliable and close to those obtained from systematic reviews. There is empirical evidence that rapid synthesis increases challenges with respect to robustness and that the responder will be challenged about health policy and system issues [19]. Nonetheless, conclusions between systematic reviews and rapid synthesis were generally consistent [31]. Future investigation may detect the impact of these shortcuts on the validity of our proposal and its importance on decision-making.

A previous Delphi study obtained consensus about core principles of rapid evidence synthesis methods [32]. Our proposal meets the main requirements [33]: a decision maker will be endorsing the methods and the timelines of the synthesis (less time than a systematic review), feasible shortcuts, and transparent reporting. Such products will support HTA decisions, so vigilance in regard to their quality is necessary and must be included in the editorial routine [34].

In comparison with others methods of rapid reviews [8], our proposal is distinguished by the use of GRADE core principles in the synthesis [23]. This may reflect that the panel is updated with new approaches.

Other consensus methods include the nominal group technique and the consensus conference [20]. To the best of our knowledge, these strategies were not adopted to obtain accordance in rapid evidence synthesis. Probably, HTA organizations consider the expensiveness of these processes and the risk of poor direction of discussions.

Rapid evidence synthesis has become extensively adopted by HTA agencies in response to the real-world appeal of evidence-based data to support decisions [9]. If systematic reviews or HTA reports are long-delayed and pricey [10], there will be no standard methods of rapid response [1]. The best approach needs to be resolved based on the feasible evidence, time pressure, contextual issues, and the urgency of decision-makers [35]. This work provides insight into the perspective of Brazilian experts, finding that the integrity of the response producer, purpose of key questions, and acceptable methodological trade-offs are particularly important factors.

## Conclusions

Systematic reviews are very hard to progress in health managers setting. We reached feasible shortcuts to obtain valuable and secure information by rapid response. The guidelines for rapid response in HTA may be useful for the health system managers to make decisions, including the Brazilian Ministry of Health. These standards were sustained by a formal consensus of Brazilian experts in HTA. The adoption of our methodological process should improve both the quality and consistency of HTA rapid decisions. We encourage research on the validity, acceptability and practicality of these methods, as well as an analogous analysis in another context to compare the results.

## Abbreviations

AMSTAR: Assessing the Methodological Quality of Systematic Reviews
ANVISA: Brazilian National Health Surveillance Agency
GRADE: Grading of Recommendations Assessment, Development and Evaluation
HTA: Health technology assessment
PICO: Population, Intervention, Comparison, Outcome
QUADAS: Quality assessment of studies of diagnostic accuracy included in systematic reviews
ROBIS: Risk of bias in systematic reviews
SUS: Unified Brazilian Health System
